# Comparison between the Therapeutic Effect of Metformin, Glimepiride and Their Combination as an Add-On Treatment to Insulin Glargine in Uncontrolled Patients with Type 2 Diabetes

**DOI:** 10.1371/journal.pone.0087799

**Published:** 2014-03-10

**Authors:** Cheol-Young Park, Jun Goo Kang, Suk Chon, Junghyun Noh, Seung Joon Oh, Chang Beom Lee, Sung Woo Park

**Affiliations:** 1 Department of Endocrinology and Metabolism, Sungkyunkwan University School of Medicine, Kangbuk Samsung Hospital, Jongro, South Korea; 2 Department of Endocrinology and Metabolism, Hallym University School of Medicine, Hallym University Sacred Heart Hospital, GyeongGi, South Korea; 3 Department of Endocrinology and Metabolism, Kyung Hee University School of Medicine, Seoul, Republic of Korea; 4 Department of Endocrinology and Metabolism, Inje University, Ilsan Paik Hospital, Goyang, Republic of Korea; 5 Department of Endocrinology and Metabolism, Hanyang University School of Medicine, Seoul, Korea; Glaxo Smith Kline, Denmark

## Abstract

**Aims:**

To compare the commonly prescribed oral anti-diabetic drug (OAD) combinations to use as an add-on therapy with insulin glargine in patients with uncontrolled type 2 diabetes despite submaximal doses of OADs.

**Methods:**

People with inadequately controlled type 2 diabetes (n = 99) were randomly assigned on a 1∶1∶1 basis to receive insulin glargin, with fixed doses of glimepiride, metformin, and glimepiride plus metformin. Outcomes assessed included HbA1c, the changes in fasting glucose levels, body weight, serum lipids values, insulin dose and symptomatic hypoglycemia.

**Results:**

After 24 weeks, HbA1C levels improved from (mean ± SD) 8.5±0.9% to 7.7±0.8% (69.0±10.0 mmol/mol to 60.8±8.6 mmol/mol) with insulin glargine plus metformin, from 8.4±1.0% to 7.7±1.3% (68.8±10.6 mmol/mol to 61.1±14.4 mmol/mol) with insulin glargine plus glimepiride and from 8.7±0.9% to 7.3±0.6% (71.7±9.8 mmol/mol to 56.2±6.7 mmol/mol) with insulin glargine plus glimepirde plus metformin. The decrease in HbA1c was more pronounced with insulin glargine plus glimepiride plus metformin than with insulin glargine plus metformin (0.49% [CI, 0.16% to 0.82%]; P = 0.005) (5.10 mmol/mol [CI, 1.64 to 8.61]; P = 0.005) and insulin glargine plus glimepiride (0.59% [CI, 0.13% to 1.05%]; P = 0.012) (5.87 mmol/mol [CI, 1.10 to 10.64]; P = 0.012) (overall P = 0.02). Weight gain and the risk of hypoglycemia of any type did not significantly differ among the treatment groups.

**Conclusion:**

The combination therapy of metformin and glimepiride plus glargine insulin resulted in a significant improvement in overall glycemic control as compared with the other combinations.

**Trial registration information:**

ClinicalTrials.gov, NCT00708578.

The approval number of Kangbuk Samsung hospital's institutional review board (IRB): C0825.

## Introduction

Diabetes is an increasing global health problem and the proportion of people with type 2 diabetes has increased in a much shorter time, throughout Asia [1]–[3]. UKPDS found that intensive glucose control starting at the time of diagnosis is associated with a significantly decreased risk of coronary events, in addition to the well-established reduction in the risk of microvascular disease [4]. Therefore, tight glycemic control is of great importance in type 2 diabetes. This has led to several guidelines recommending the early addition of basal insulin therapy in patients who do not meet target HbA1C levels [5]. Although addition of a third oral agent can be considered, especially if the HbA1C level is close to target goal, this approach is usually not preferred, as it is no more effective in lowering glycemia, and is more costly, than initiating or intensifying insulin [5], [6].

Glargine insulin is a peakless, long-acting insulin analog that provides 24-h basal insulin replacement and achieve target HbA1C level with less hypoglycemia in most patients when it is added to existing OADs as a once-daily injection [7]–[13]. However, it is not clear that the combination therapy of glargine insulin and which OADs is effective and safe in reducing fasting glucose and HbA1C among type 2 diabetes mellitus with chronic hyperglycemic control. And there is little evidence to support optimal combination treatment with basal insulin and OADs especially in the Asian population.

The number of available OADs has increased significantly in the last decade, but sulfonylurea, metformin and their combination are still frequently prescribed class of OADs. Therefore many clinical trials also investigated the therapeutic effect of insulin glargine as an add-on therapy to different OAD treatments in uncontrolled patients with diabetes. However, the previous studies showed the various range of the efficacy and safety of those treatments. The inconsistency of existing data might be due to multiple reasons including the various duration of diabetes, the range of age, the size of population and indirect comparison between different OADs.

Therefore the current study aims to compare the commonly prescribed OADs as an add-on therapy of insulin glargine in Korean patients with uncontrolled type 2 diabetes even with submaximal doses of metformin and sulfonylurea as the combination therapy.

## Patients and Methods

### Study design

Our study was a 28-week, open-label, randomized, controlled, multicenter, parallel-group clinical trial, conducted in Korea. Patients were enrolled from outpatient clinics of 5 centers in Korea from 20 June 2008 to 18 December 2009. The study consisted of a screening/titration phase of up to 4 weeks and a 24-week treatment phase. Patients with type 2 diabetes who did not achieve good metabolic control while receiving nearly maximal dose of oral anti-diabetic drugs for 4 weeks were randomly assigned to receive additional treatment for 24 weeks with insulin glargine in the morning with fixed doses of glimepiride, metformin or glimepiride plus metformin. With a randomization schedule generated by the Clinical Research Organization eligible patients were linked sequentially to treatment codes allocated at random. This schedule was stratified by center on a 1∶1∶1 basis. Blocked randomization was performed with a block size of 3, 6 with blinded treatment allocation by SAS program.

The primary efficacy objective of this study was to compare glycemic control (measured by HbA1C) among glimepiride, metformin and glimepiride plus metformin with add-on therapy of glargine in type 2 diabetes mellitus receiving nearly maximal combination therapy with metformin plus sulfonylurea for chronic hyperglycemic control. The HbA1C assay was measured by high-performance liquid chromatography (Bio-Rad Diamat, Munich, Germany) in the central laboratory (INTERLAB, Munich, Germany); the reference range was 25 to 43 mmol/mol (4.4% to 6.1%). Samples were taken for HbA1C assessment in screening period and at weeks 4, 8, 16, 24 or at study discontinuation. Secondary efficacy variables included the incidence of symptomatic hypoglycemia, the percentage of subjects who reached HbA1c<7.0 or 7.5% at the last time and the changes in fasting glucose levels, body weight, and serum lipids values.

The protocol for this trial and supporting CONSORT checklist are available as supporting information; see Checklist S1 and Protocol S1.

### Ethics statement

The study was conducted in accordance with the Declaration of Helsinki and its modifications. The institutional review board of Kangbuk Samsung hospital, Hallym University Sacred Heart Hospital, Kyunghee university medical center, Ilsan Paik Hospital and Hangyang university Guri hospital reviewed and approved the study protocol and all patients gave written informed consent for participation before entry into the trial.

### Study populations

This study included men and women 18 to 80 years old who had had type 2 diabetes for at least 6 months, with a BMI of <30 kg/m^2^, HbA1C levels ≥7.0% to ≤11.0% (≥53 to ≤97 mmol/mol) despite ≥3 months of treatment with a stable dose of both a sulfonylurea (more than daily 4 mg glimepiride or equivalent to dose of other sulfonylureas) and metformin (at least 1000 mg/day) up to the maximum tolerated dose) and fasting serum C-peptide >0.99 ng/mL. Patients were also required to have a demonstrated ability and willingness to inject insulin and to perform self-monitoring of blood glucose (SMBG) with use of a plasma-referenced glucose meter. Exclusion criteria included type 1 diabetes; insulin treated type 2 diabetes or having previously received long-term insulin; history of hypersensitivity to the investigational product or to drugs with similar chemical structures; levels of alanine aminotransferase or aspartate aminotransferase greater than twice the upper limit of the normal range; a creatinine level greater than 1.5 mg/dL in man and 1.4 mg/dL in woman; pregnancy or lactation; treatment of systemic treatment with corticosteroids within 3 months; and patients unable to perform SMBG or inject insulin on their own.

### Study intervention

During the treatment phase, patients had to provide daily self-measured fasting blood glucose values, and episodes of hypoglycemia were recorded in a standardized diary. The investigators checked these values, and the insulin dose was adjusted according to a predefined titration regimen.

When combination therapy (insulin+OADs) was initiated, sulfonylurea and metformin doses remained unchanged during the treatment phase of the study. Subjects were randomized on a 1∶1∶1 basis to receive insulin glargine (LANTUS® SoloSTAR®, sanofi-aventis) in the morning with fixed doses of glimepiride (Amaryl® 4 mg/day), metformin (Glucophage®XR 1500 mg/day) and glimepiride(Amaryl® 4 mg/day) plus metformin (Glucophage®XR 1500 mg/day). Insulin glargine was administered as a single daily subcutaneous injection in the morning at a starting dose of 0.2 U/kg, sometimes 10 IU/day for 3 days, which was titrated every third day to achieve a target FPG value of 5.0–7.2 mmol/l (90–130 mg/dL). Study medication accountability logs for insulin glargine, glimepiride and metformin were collected and evaluated for assessment of compliance.

### Safety measures

Safety was assessed in the safety population through adverse events, hypoglycemia, body weight, physical examinations, vital signs, and blood chemistry Physical examinations and vital signs were measured at each visit. Capillary blood glucose, hypoglycemic episodes, and adverse events were documented by the patient via diary cards or were recorded by the study investigator when mentioned by the patient. Hypoglycaemia was defined as either symptomatic or asymptomatic in the context of a glucose level ≤3.3 mmol/l (≤60 mg/dL). Severe hypoglycemia was defined as an event with symptoms consistent with hypoglycemia, associated with a BG level ≤2.0 mmol/l (≤36 mg/dL) or with prompt recovery after oral carbohydrate, intravenous glucose or glucagon administration, and the requirement of third party assistance. Nocturnal hypoglycemia was defined as hypoglycemia that occurred while the patient was asleep, and before getting up in the morning.

Adverse events (AEs) were also recorded by the investigator. Causality was determined by the investigator and categorized as possibly related or not related to the study medication.

### Sample size calculation

When the sample size in each group is at least 5, a 5% significance level two-sided ANOVA (Analysis of Variance) test for equality of HbA1c will have 90% power to detect difference between three groups assuming that common standard deviation is 0.1 and variance of the three individual group means is 0.011. If the ANOVA test showed significant difference among three groups, multiple comparison (1 vs. 2, 2 vs. 3 and 1 vs. 3) would be conducted. Based on the assumption that a common standard deviation is 0.1 and the pairwise difference is at least 0.1, 29 patients per group would be required to detect difference using t-test with a Bonferroni adjustment of α = 0.05/3 and 90% power. Considering drop out rate of 10%, the total sample size would be at least 99.

### Statistical methods

The primary end point assessed in the intention-to-treat (ITT) population was HbA1C at 28 weeks, using ANCOVA adjusted for baseline. The last observation carried forward (LOCF) principle was used in patients dropped out before 28 weeks. In pairwise comparison among 3 groups, a P value of 0.05/3 (equivalent to a Bonferroni-adjusted [3 tests] P value of 0.05) was considered significant. The percentage of patients who reached HbA1C<7.0 or 7.5% at the end of the trial and the incidence of symptomatic hypoglycemia during the last month of treatment were analyzed using chi-square test or Fisher's exact test (for small numbers of cell count). The safety population includes all randomized subjects who received study product. Additional efficacy and safety assessments such as FPG, lipid profile and change in weight after 28 weeks were also analyzed with ANCOVA adjusted for baseline.

All data were presented as mean ± SD and values at p<0.05 were considered statistically significant. All statistical analyses were performed with statistical analysis software for PC (SAS 9.1.3, SAS Institute Inc., SAS Campus Drive, Cary, North Carolina 27513, USA).

## Results

### Patients studied

**Figure 1:**
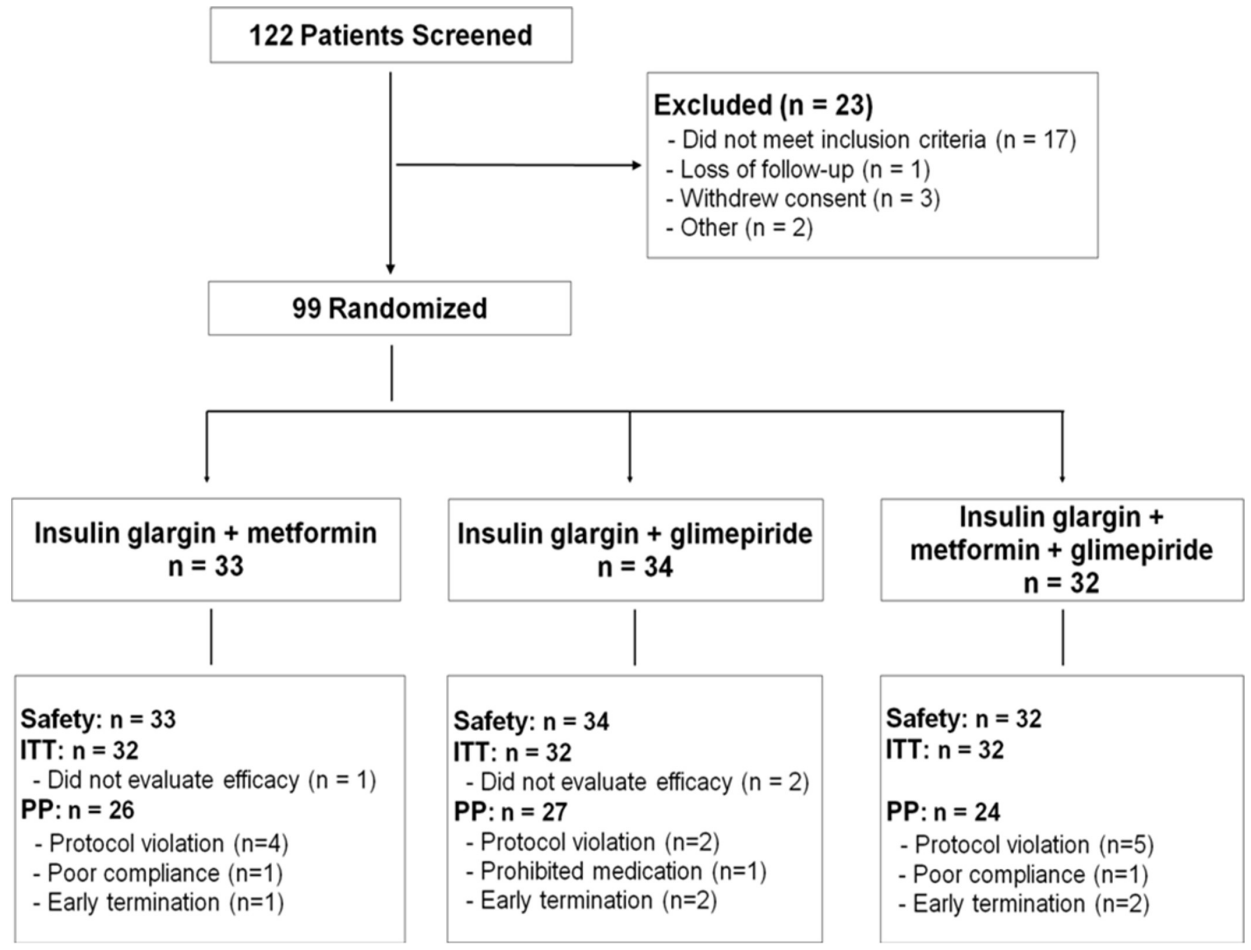
Flow diagram of this study. Abbreviations are; ITT, intent-to-treat; PP, per protocol.

The progression of this study from screening to study endpoint is summarized in Figure 1. Of the 99 patients randomized, 33 were randomized to the insulin glargine plus metformin group, 34 to the insulin glargine plus glimepirde group and 32 to the insulin glargine plus glimepirde plus metformin group. The PP, ITT and safety populations comprised 77, 96 and 99 patients, respectively. The baseline characteristics of the study population indicated no significant difference between the groups, both for clinical and laboratory characteristics (Table 1).

**Table 1:** Baseline clinical and biochemical characteristics of the study subjects.*

	Insulin glargine + metformin	Insulin glargine + glimepiride	Insulin glargine + metformin+glimepiride	P value
	33	34	32	
Age (years)	55.8±10.5	57.3±9.2	56.8±10.9	0.82171)
Sex (% male)	20(60.61)	20(58.82)	23(71.88)	0.49412)
BMI (kg/m^2^)	25.1±3.6	25.6±2.7	25.2±2.7	0.80161)
Waist (cm)	89.4±10.4	89.7±6.4	90.7±6.9	0.79141)
Duration of diabetes (years)	11.3±6.4	13.0±8.0	11.7±5.0	0.53691)
Chronic diabetes complication, n (%)	15(45.45)	16(37.06)	11(34.38)	0.52942)
HbA1c (mmol/mol)	69.0±10.0	68.8±10.6	71.7±9.8	0.40531)
(%)	8.4±0.9	8.4±1.0	8.7±0.9	0.44971)
FPG (mmol/L)	9.0±2.8	8.9±3.2	9.2±2.4	0.94091)
Fasting plasma C-peptide (mmol/L)	0.8±0.5	0.8±0.4	0.8±0.4	0.95541)
TC (mmol/L)	4.1±0.9	3.8±0.8	3.9±0.9	0.44501)
TG (mmol/L)	1.7±1.2	1.5±0.8	1.5±1.1	0.70841)
HDL-C (mmol/L)	1.1±0.3	1.1±0.3	1.1±0.3	0.81791)
LDL-C (mmol/L)	2.2±0.8	2.1±0.7	2.1±0.7	0.71001)

*Plus-minus values are means±SD.

The body-mass index (BMI) is the weight in kilograms divided by the square of the height in meters. BP, blood pressure; FPG, fasting plasma glucose; TC, total cholesterol; TG, Triglyceride; HDL-C, High density lipoprotein cholesterol; LDL-C, Low density lipoprotein cholesterol.

1) ANOVA,

2) chi-square test.

### Glycemic control

#### HbA1C

**Figure 2:**
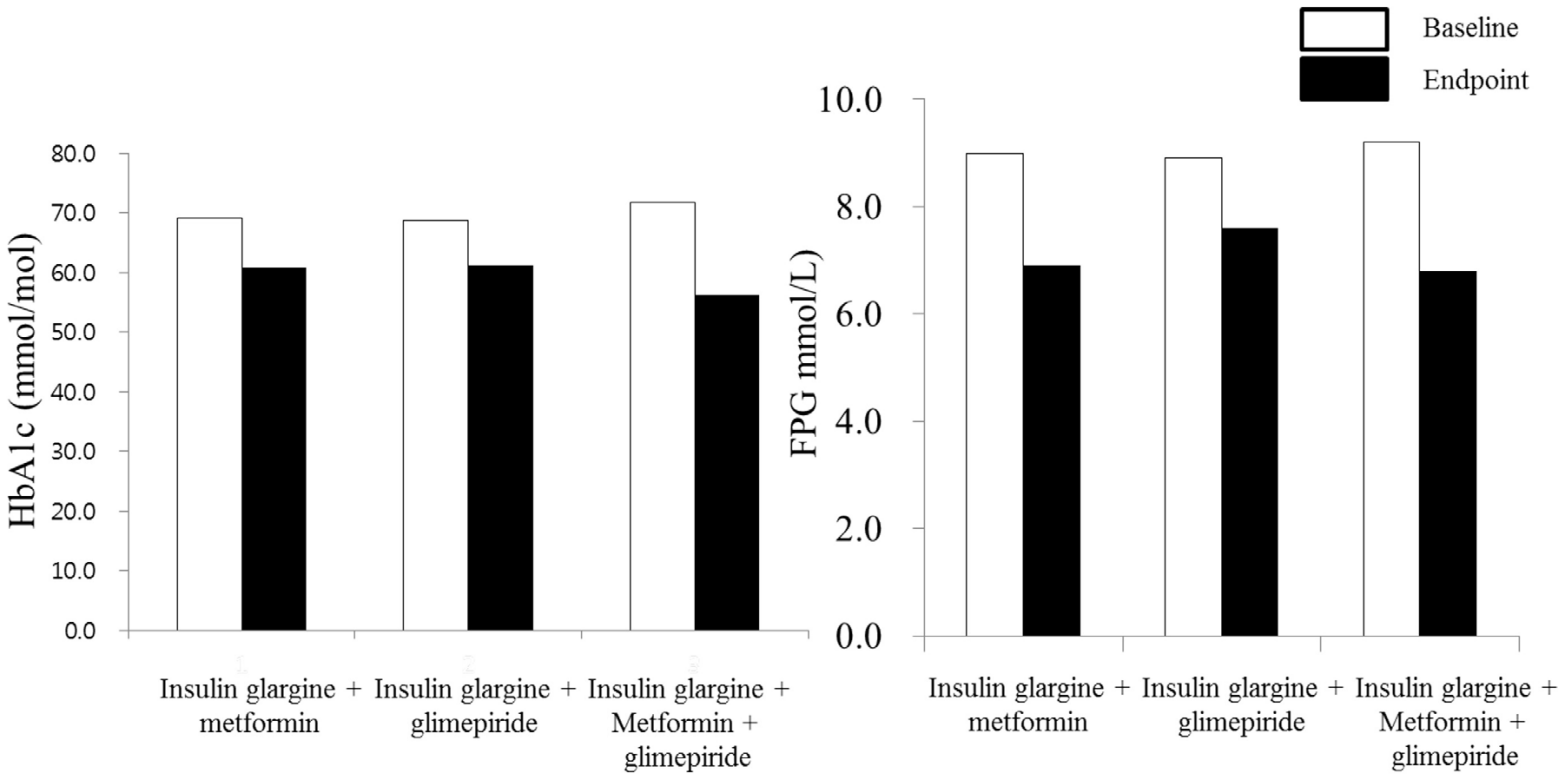
Reduction in mean HbA1C(A) and FPG(B) from Baseline to end of study. Abbreviations are; FPG = Fasting plasma glucose.

Over the 24-week treatment period, HbA1C levels improved from 8.5±0.9% to 7.7±0.8% (69.0±10.0 mmol/mol to 60.8±8.6 mmol/mol) with insulin glargine plus metformin, from 8.4±1.0% to 7.7±1.3% (68.8±10.6 mmol/mol to 61.1±14.4 mmol/mol) with insulin glargine plus glimepirde and from 8.7±0.9% to 7.3±0.6% (71.7±9.8 mmol/mol to 56.2±6.7 mmol/mol) with plus insulin glargine plus glimepirde plus metformin (Figure 2 A). HbA1C levels improved by −0.75 (two-sided 95% CI, −1.14% to 0.36%) [−8.20 mmol/mol (two-sided 95% CI, −12.45 to −3.95)] with insulin glargine plus metformin, −0.70 (CI, −1.15% to −0.26%) [−7.69 mmol/mol (two-sided 95% CI, −12.52 to −2.86)] with insulin glargine plus glimepirde, and −1.41 (CI, −1.67% to −1.16%) [−15.47 mmol/mol (two-sided 95% CI, −18.31 to −12.64)] with insulin glargine plus glimepirde plus metformin. Improvement in HbA1c was significantly higher with insulin glargine plus glimepirde plus metformin than with insulin glargine plus metformin (0.49% [CI, 0.16% to 0.82%]; P = 0.005) (5.1 mmol/mol [CI, 1.64 to 8.61]; P = 0.005) and insulin glargine plus glimepirde (0.59% [CI, 0.13% to 1.05%]; P = 0.012) (5.87 mmol/mol [CI, 1.10 to 10.64]; P = 0.012) (overall P = 0.02) (Table 2). The participants measured by HbA1C level less than 6.5% over the study duration did not differ among the groups. However, more patients reached an HbA1c level of 7.5% or less with insulin glargine plus glimepirde plus metformin (24 of 32 [75.0%]) than with insulin glargine plus metformin (14 of 33 [43.8%]; *P* = 0.0109) (Table 2).

**Table 2:** Effect of insulin glargin plus metformin, insulin glargin plus glimepirde or insulin glargin plus glimepirde plus metformin on Glycemic Control.

	Insulin glargine + metformin	Insulin glargine + glimepiride	Insulin glargine + metformin+glimepiride	Overall P value
Patients, n	32	32	32	
Mean improvement in HbA1c level† (95% CI), %	−0.75 (−1.14, −0.36)	−0.7‡ (−1.15, −0.26)	−1.41§ (−1.67, −1.16)	0.02
HbA1c level <7.0%, n (%)	5(15.6)	10∥ (31.3)	11¶ (34.4)	0.1949
HbA1c level <7.5%, n (%)	14(43.8)	18# (56.3)	24± (75.0)	0.0385

† From end point to baseline.

‡ P = 0.2 versus Insulin glargine + metformin.

§ P = 0.005 versus Insulin glargine + metformin and P = 0.012 versus Insulin glargine + glimepiride.

∥ P = 0.2 versus Insulin glargine + metformin.

¶ P = 0.021 versus Insulin glargine + metformin and P = 0.017 versus Insulin glargine + glimepiride.

# P = 0.3137 versus Insulin glargine + metformin.

± P = 0.0109 versus Insulin glargine + metformin and P = 0.1143 versus Insulin glargine + glimepiride.

#### Fasting blood glucose

Fasting blood glucose levels also improved in all three groups: from 9.0±2.8 to 6.9±2.3 mmol/l (162.5±49.9 mg/dL to 124.4±41.6 mg/dL) with insulin glargine plus metformin, from 8.9±3.3 to 7.6±2.7 mmol/l (160.3±59.1 mg/dL to 136.1±48.8 mg/dL) with insulin glargine plus glimepirde, and from 9.2±2.4 to 6.8±1.3 mmol/l (165.1±42.7 mg/dL to 121.6±22.5 mg/dL) with insulin glargine plus glimepirde plus metformin (Figure 2 B). The average reduction in fasting blood glucose level achieved over the study duration did not differ among the groups (for all groups, *P* = 0.1607).

### Adverse events

AEs were assessed in the Safety population. The number of adverse events during the treatment phase was similar in all three treatment groups (insulin glargine plus metformin, 12; insulin glargine plus glimepiride, 16; insulin glargine plus metformin plus glimepiride, 11). There was no report of withdrawals or treatment-emergent adverse events related to the study medication, during the treatment phase. Serious treatment-emergent adverse events were reported by 5 patients but were not related to the study medications.

The risk of hypoglycemia of any type was comparable and the number of patients experiencing episodes of hypoglycemia was similar among all treatment (Table 3). There was no difference in the frequency of clinically noteworthy abnormal laboratory values among the treatment groups (data not shown).

**Table 3:** Hypoglycemic episodes in study subjects treated with insulin glargine plus metformin, insulin glargine plus glimepirde or insulin glargine plus glimepirde plus metformin.

	Insulin glargine + metformin		Insulin glargine + glimepiride		Insulin glargine + metformin+glimepiride		P value
	n(%). [# of episode]	Rate*	n(%). [# of episode]	Rate*	n(%). [# of episode]	Rate*	
Patients, n	33		34		32		
All episode of hypoglycemia	15(45.5).[64]	4.1	20(58.8).[95]	5.8	17(53.1).[83]	5.1	0.5469^a^
All episode of asymptomatic hypoglycemia	8(24.24).[19]	1.2	10(29.41).[11]	0.7	7(21.88).[33]	2.0	0.7700^a^
All episode of symptomatic hypoglycemia	12(36.36)[45]	2.9	14(41.18).[84]	5.1	15(46.88).[50]	3.1	0.6904^a^
Nocturnal hypoglycemia	3(9.09)[8]	0.5	6(17.65).[13]	0.8	3(9.38).[8]	0.5	0.5832^b^
Severe hypoglycemia	0 (0.00)		0 (0.00)		0 (0.00)		-

* Rate: event rate per patient year (calculated as N events/Total patient-years).

a Chi-square test.

b Fisher's exact test.

### Changes of metabolic parameters

Weight gain during the treatment period was 1.26±2.60 kg with insulin glargin plus glimepiride and 1.38±2.97 kg with insulin glargin plus metformin plus glimepiride. However, the weight gain did not significantly differ among the treatment groups (*P*>0.4). The lipid profiles and blood pressure also did not significantly differ among the treatment groups (data not shown).

### Insulin dose

**Figure 3:**
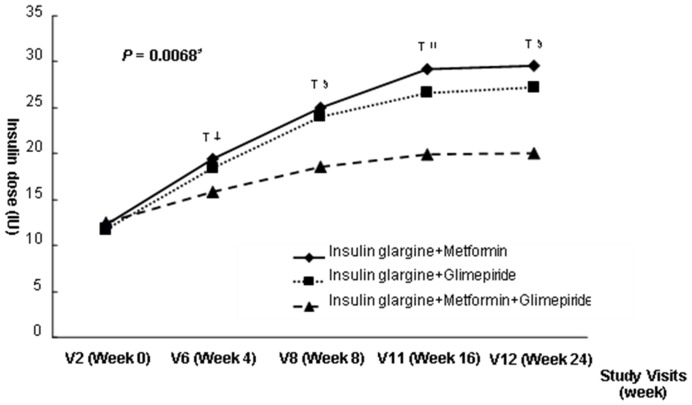
Mean insulin dose from baseline to the end of study. *Repeated measure of ANOVA. ^†^P<0.05 versus Insulin glargin + glimepiride. ^‡^P<0.05 versus Insulin glargin + metformin. ^§^P<0.01 versus Insulin glargin + metformin. ^ll^P<0.001 versus Insulin glargin + metformin.

The insulin doses increased from a mean (±S.D.) of 12.2±2.7 IU to 29.5±13.3 IU for insulin glargine plus metformin, from 11.8±2.0 to 27.2±14.2 U for insulin glargine plus glimepirde, and from 12.5±2.5 to 20.1±10.3 U for insulin glargine plus glimepirde plus metformin, over the 24-week treatment period. Initial insulin doses were similar in the three treatment groups. However, the change of the insulin dose from baseline to end point significantly differed among the groups. (Figure 3).

## Discussion

The results of this study demonstrated that after 24-weeks of treatment, the combination therapy of metformin and glimepiride plus glargine insulin in patients failing combination therapy with sub-maximal doses of OADs resulted in significant improvement in glycemic control compared with the combination therapy of metformin or sulfonylurea monotherapy plus glargine insulin without differential increase in the risk of weight gain and hypoglycemia and suggest that the addition of insulin glargine to glimepiride and metformin combination therapy in patients with inadequately controlled type 2 diabetes is an effective treatment strategy for achieving glycemic control.

The ADA and EASD guidelines and treatment algorithm for the most appropriate intervention in the management of hyperglycemia for patients with T2DM emphasized that early addition of insulin therapy in patients who do not meet target goals is the most effective of diabetes medications in lowering glycemia [5]. Addition of insulin glargine to glimepiride or metformin monotherapy is one of effective treatment strategies for achieving glycemic control. In this study, after 24 weeks, mean changes from baseline HbA1C was similar between insulin glargine plus metformin and insulin glargine plus glimepirde.

Previous controlled trials comparing the combination therapy with oral hypoglycemic agents (SU plus Metformin) and insulin therapy in patients with poorly controlled type 2 diabetes are sparse [14]–[16], and among these studies, reports about a peakless, long-acting insulin analog are executive. In a study by Yki JH et al [16], 96 patients with type 2 diabetes to 1 year of treatment were randomized to receive bedtime insulin plus glyburide and placebo, metformin and placebo, glyburide and metformin, or a second injection of insulin and were evaluated with their effects on weight gain, frequency of hypoglycemic episodes, and glycemic control in patients with type 2 diabetes whose disease was inadequately controlled with sulfonylurea therapy alone. This study demonstrated that combination therapy with bedtime insulin plus metformin prevents weight gain and this regimen also seems superior to other bedtime insulin regimens with respect to improvement in glycemic control and frequency of hypoglycemia. However, our study showed the optimal oral anti-diabetic drugs combinations to add-on therapy of insulin glargine in patients with uncontrolled type 2 diabetes despite sub-maximal doses of OADs (SU+metformin) which are most frequently available among patients with uncontrolled type 2 diabetes in real practice. In the present study, we expected patients receiving both oral drugs (glimepiride and metformin) in addition to insulin glargine to require greater improvements in HbA1C, greater decreases in their insulin doses, greater increases in hypoglycemic episodes and weight than those receiving insulin glargine plus only one oral drug (glimepiride or metformin). It could be argued that it is inappropriate to compare measures of glycemic control among the different groups given the different insulin doses. On the other hand, the dose adjustments were made by clinician through the protocol for glycemic control and were the result of a randomized trial. However, the risk of weight gain and hypoglycemia did not significantly differ among the treatment groups. The reasons for this remain uncertain, but we suggest that combination therapy of metformin plus glimepiride with add-on therapy of insulin glargine lead a decrement of insulin doses without differential increase in the risk of hypoglycemia.

In the present study, the participants measured in HbA1C level of 7.0% or less over the study duration did not differ among the groups. Most of the clinical practice guidelines for glycemic control target of type 2 diabetes since early the 2000s have recommend the achievement of near-normoglycemia to prevent diabetes related vascular complications and glycemic goals of HbA1C≤7.0% (53 mmol/mol) should be attained in non-pregnant young type 2 diabetes mellitus without severe complications or hypoglycemia[17], [18]. However, three recently published large RCT studies (ACCORD, ADVANCE, VADT) have demonstrated no significant benefits in cardiovascular outcome with intensive glycemic control in type 2 diabetes [19] and participants in the ACCORD and ADVANCE trials and the VADT had type 2 diabetes mellitus for 8.0 to 11.5 years. As a result, the glycemic target range for patients with type 2 diabetes mellitus should be individualized according to age; stage of disease, both in terms of duration and presence of macro- and microvascular complications; and propensity for hypoglycemia [20], [21]. Participants in our study had type 2 diabetes mellitus for mean 12.0 years and in the practical points, more patients reached an HbA1c level of 7.5% or less [22] with insulin glargine plus glimepirde plus metformin than with insulin glargine plus metformin.

The limitation of the present study is that the long term effects and safety of glimepiride plus metformin with add-on therapy of insulin glargine cannot be evaluated. However, this study is valuable in that it is the only study to our knowledge that evaluates the optimal OADs combination to add-on therapy of insulin glargine in patients with uncontrolled type 2 diabetes despite submaximal doses of combination therapy with metformin plus sulfonylurea. In conclusion, the combination therapy of metformin and glimepiride plus glargine insulin in patients failing combination therapy with sub-maximal doses of OADs resulted in significant improvement in glycemic control compared with the combination therapy of metformin plus glargine insulin or sulfonylurea plus glargine without differential increase in the risk of weight gain.
